# Impact of Species and Tissue Differences on In Vitro Glucuronidation of Diclofenac

**DOI:** 10.3390/molecules29245867

**Published:** 2024-12-12

**Authors:** Eric Asare, Shalom Emmanuel, Ting Du, Huan Xie, Dong Liang, Song Gao

**Affiliations:** Department of Pharmaceutical Science, College of Pharmacy and Health Sciences, Texas Southern University, 3100 Cleburne Street, Houston, TX 77004, USA; e.asare2673@student.tsu.edu (E.A.); s.emmanuel2414@student.tsu.edu (S.E.); du.ting@tsu.edu (T.D.); huan.xie@tsu.edu (H.X.); dong.liang@tsu.edu (D.L.)

**Keywords:** diclofenac, glucuronidation, species differences, tissue differences, UGT

## Abstract

Background: The aim of this study is to determine the impact of species and tissue differences on the glucuronidation of diclofenac in vitro. Method: Microsomes from different species (rat, monkey, mouse, dog, and human) and rat and human tissues (liver, intestine, and kidney) were used to assess the rate of glucuronidation reaction of diclofenac. The metabolites were quantified using ultra high-performance liquid chromatography (UHPLC) and fitted into a Michaelis–Menten model to determine the metabolic kinetic parameters. Results: The results showed higher rates of metabolism in the liver as compared to that of the intestine and kidney by both human and rat tissues microsomes. There were also differences in the rate of metabolism in the liver across the tested species, with mouse liver microsome having the highest maximum reaction rate (V_max_) at 7.22 nmol/min/mg followed by human liver microsome at 6.66 ± 0.33 nmol/min/mg, dog liver microsome at 5.05 ± 0.42 nmol/min/mg, monkey liver microsome at 3.88 ± 0.15 nmol/min/mg, and rat liver microsome at 0.83 ± 0.04 nmol/min/mg. Conclusions: This study demonstrated that the liver is the major organ for the glucuronidation of diclofenac. In addition, glucuronidation of diclofenac was different across the tested species; therefore, the influence of species should be taken into consideration in the pharmacological, pharmaceutical, and toxicological study of diclofenac.

## 1. Introduction

Diclofenac is a widely prescribed non-steroidal anti-inflammatory drug (NSAID) that has promising anti-inflammatory, analgesic, and antipyretic properties. It is clinical proven effective in treating various acute and chronic pain and inflammation conditions [1]. Pharmacological studies have demonstrated that diclofenac exerts its efficacy by inhibiting cyclooxygenases, thereby reducing the production of prostaglandin E2 (PGE_2_), a pro-inflammatory prostaglandin [2]. Animal models have also shown that diclofenac exhibits anti-cancer efficacy, indicating its potential in cancer treatment [3]. However, clinical studies have revealed severe side effects associated with diclofenac therapy such as bleeding, abdominal pain, stroke, heart attack, hepatotoxicity, and kidney injury [4,5,6].

**Figure 1:**
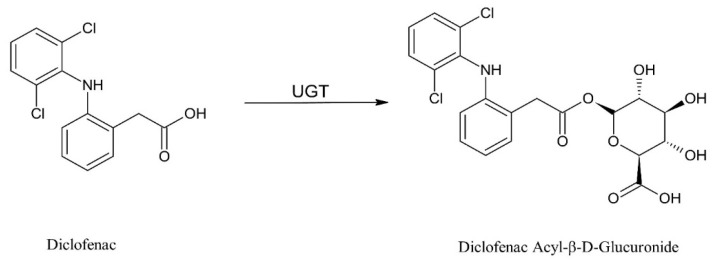
Glucuronidation mediated by uridine 5′-diphospho-glucuronosyltransferases (UGTs) to generate an acyl-glucuronide.

Diclofenac undergoes enterohepatic recirculation (EHR) via acyl glucuronidation [7,8,9], resulting in prolonged half-life in the plasma. When diclofenac is absorbed in the intestine after oral administration, a fraction of the parent drug reaches the liver through portal vein and then undergoes phase II glucuronidation mediated by uridine 5′-diphospho-glucuronosyltransferases (UGTs) to generate an acyl glucuronide (Figure 1). Then, the diclofenac acyl glucuronide can be secreted back into the gastrointestinal (GI) tract through the bile and then be hydrolyzed by microbial beta-glucuronidase in the lower small intestine (i.e., ileum) and the colon to release diclofenac for reabsorption in the GI tract to form EHR. It was reported that EHR has a significant impact on the pharmacokinetics of diclofenac administered orally [7,8,9], which may have an impact on its pharmacological efficacy and/or GI toxicity [7,10,11]. Therefore, many studies have been reported on the glucuronidation of diclofenac even though glucuronide may not be the major metabolite in humans compared to that of phase I metabolism.

Different animal models, including those of dogs, mice, rats, and monkeys, have been used in diclofenac’s pharmacokinetic, pharmacodynamic, and toxicological studies; however, the glucuronidation of diclofenac in different species has not been reported. Therefore, in this study, we investigated the glucuronidation of diclofenac across different species, including mice, rats, monkeys, dogs, and humans, using tissue microsomes. Additionally, we determined glucuronidation in the different tissues using rat and human liver, intestine, and kidney microsomes in in vitro studies to determine tissue specificity in the glucuronidation of diclofenac.

## 2. Results

### 2.1. Glucuronidation Reaction

**Figure 2:**
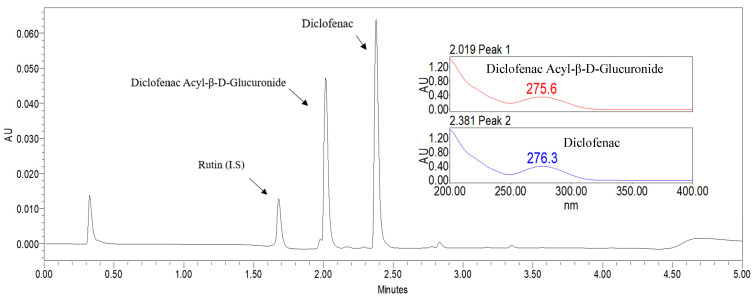
Chemical structures, a representative UHPLC chromatogram in KPI solution, and UV spectra of diclofenac and diclofenac Acyl-β-D-Glucuronide (AcD-G).

When diclofenac was incubated with phase II rat metabolic microsomes or supersomes, an additional chromatographic peak was observed in the UHPLC analysis beside the parent compound diclofenac. The retention time of the peak (2.01 min) was identical with that of diclofenac acyl glucuronide (AcD-G) standard. Additionally, the UV spectrum of the additional peak showed one absorption peak at 275.6 nm, which was similar to that of standard AcD-G. Based on this evidence, the observed metabolic peak was identified as AcD-G (Figure 2).

### 2.2. Diclofenac Glucuronidation with Different UGT Isoforms

**Figure 3:**
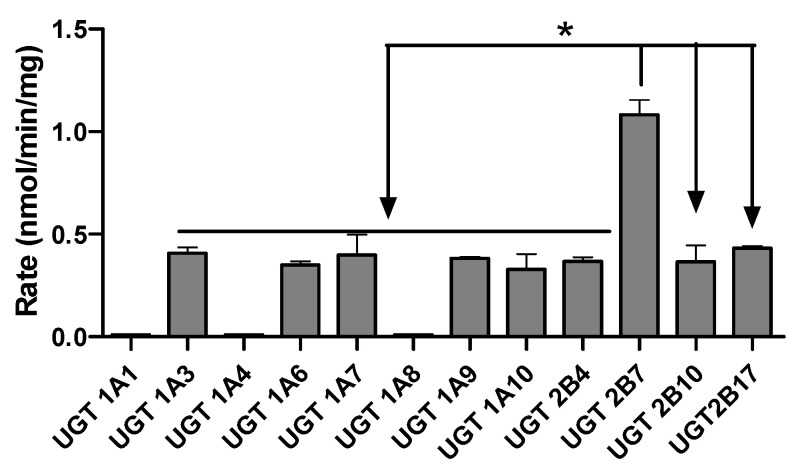
Glucuronidation of diclofenac by recombinant human UGT isoforms. Data expressed as mean ± SD (*n* = 3). * indicate significance differences across all UGTs in comparison with UGT2B7.

Following the two-hour incubation of diclofenac with various isoforms of UGT, the results obtained showed that UGT 2B7 had the highest activity in the glucuronidation of diclofenac with a reaction rate of 1.082 nmol/min/mg of protein. This was more than two-fold the reaction rate of the isoforms UGT 1A3, 1A6, 1A7, 1A9, 1A10, 2B4, 2B10, and 2B17, which had an average reaction rate of ≤0.4 nmol/min/mg. No glucuronide conjugate was detected when diclofenac was incubated with UGT1A1, 1A4, and 1A8, suggesting the activity of these three isoforms was low. Therefore, UGT2B7 was the major isoform among those tested (Figure 3).

### 2.3. Diclofenac Glucuronidation with Human and Rat Liver, Intestine, and Kidney Microsomes

**Figure 4:**
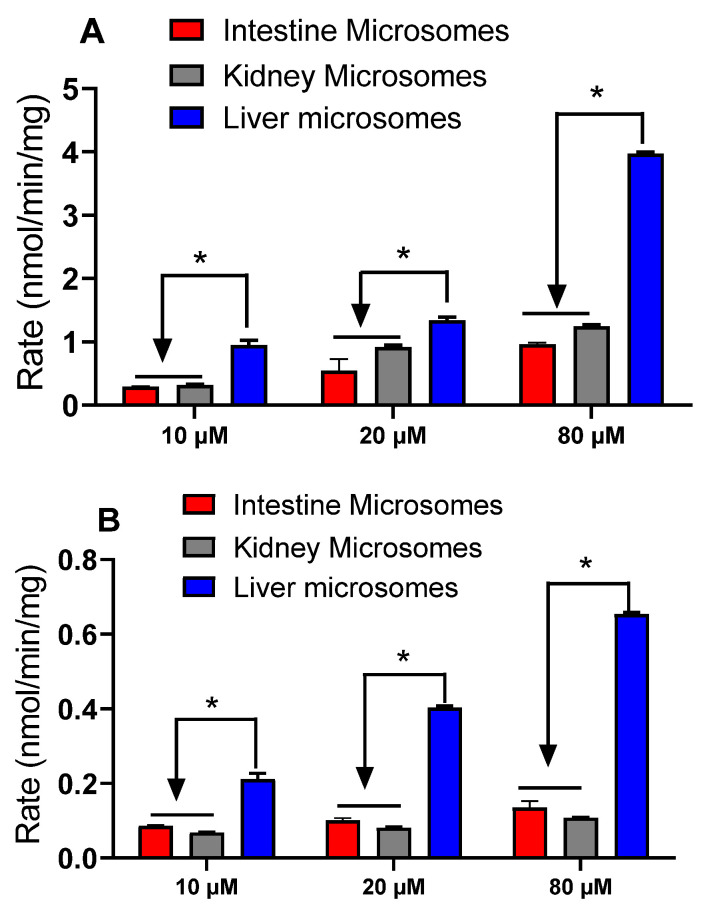
Glucuronidation of diclofenac by human (**A**) and rat (**B**) intestinal, kidney, and liver microsomes. Data expressed as mean ± SD (*n* = 3). * indicate significance differences across various concentrations at (*p* < 0.05, *t*-test).

Diclofenac glucuronide formation rates by rat and human liver, intestine, and kidney microsomes were obtained to evaluate tissue specificity. The results showed that the liver glucuronidation rates were significantly higher than those of the kidney and intestine at the tested concentrations (10 μM, 20 μM, and 80 μM) (Figure 4). These findings suggest that the liver could be the major organ metabolizing diclofenac in vivo.

### 2.4. Diclofenac Glucuronidation in Liver Microsomes of Different Species at 10 μM

**Figure 5:**
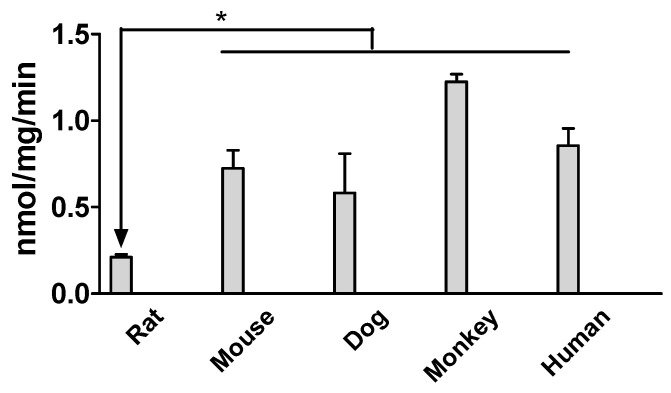
Diclofenac glucuronidation activities in mouse, rat, dog, monkey, and human liver microsomes. Data expressed as mean ± SD (*n* = 3). * indicates significance difference of other species compared to the liver of the rat at (* *p* < 0.05).

Diclofenac glucuronidation activities in the liver microsomes of humans, mice, rats, dogs, and monkeys were initially compared at a substrate concentration of 10 μM. The results showed that the metabolic rate of rat liver microsomes was the slowest at 0.21 ± 0.02 nmol/min/mg while that of monkey liver microsomes was the highest at 1.23 ± 0.04 nmol/min/mg (Figure 5). Additionally, the results indicated that the metabolic rates of monkey, mouse, and dog intestinal microsomes were comparable to that of human intestinal microsomes.

### 2.5. Kinetics of Diclofenac Glucuronidation in Liver Microsomes of Different Species

**Figure 6:**
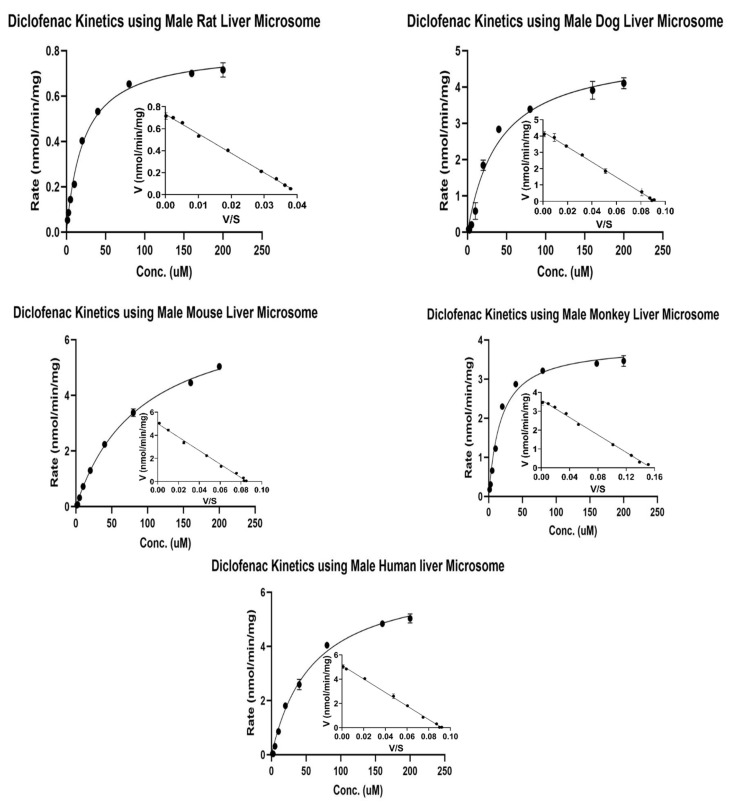
Kinetic study (V_max_) of diclofenac glucuronidation in liver microsomes of various species. Data expressed as mean ± SD (*n* = 3).

Diclofenac glucuronidation formation showed a good fit to the Michaelis–Menten model in the rat, dog, mouse, monkey, and human liver microsomes, as confirmed with Eadie–Hofstee plots, which showed a linear relationship between the reaction velocity (v) and the ratio of velocity to substrate concentration (v/[S]) within the experimental conditions (Figure 6, Table 1). The V_max_ values of these species showed an 8.7-fold difference in the order of 0.83 ± 0.04 nmol/min/mg for rat, 3.88 ± 0.15 nmol/min/mg for monkey, 5.05 ± 0.42 nmol/min/mg for dog, 6.66 ± 0.33 nmol/min/mg for human, and 7.22 ± 0.28 nmol/min/mg for mouse liver microsomes. The K_m_ values showed a 5.13-fold difference in the order of 17.90 ± 2.59 μM for monkey, 24.03 ± 4.26 for rat, 41.45 ± 10.21 μM for dog, 59.5 ± 7.79 μM for human, and 91.85 ± 8.05 μM for mouse liver microsomes. The Cl_int_ (V_max_/K_m_) values showed a 7.3-fold difference in the order of 0.03 ± 0.01 µL/min/mg for rat, 0.08 ± 0.01 µL/min/mg for mouse, 0.12 ± 0.02 µL/min/mg for human, 0.13 ± 0.01 µL/min/mg for dog, and 0.22 ± 0.01 µL/min/mg for monkey microsomes.

## 3. Discussion

Diclofenac is an approved anti-inflammatory drug, and numerous preclinical and clinical studies have been conducted investigating its pharmacological and toxicological properties. Pharmaceutical studies have shown that diclofenac undergoes glucuronidation metabolism to form an acyl glucuronide using a microsomal reaction [12]. Additionally, acyl glucuronide was identified as the major metabolites in animal and human studies [13,14,15,16]. However, the current literature on diclofenac glucuronidation lacks a comprehensive comparison among different tissues like those of the rat and human liver, intestine, and kidney; UGT isoforms; and especially species involved in this biotransformation process. In this study, we confirmed that UGT2B7 was the major isoform to metabolize diclofenac into its glucuronide. We also found that the liver had a significantly higher maximum velocity (V_max_) of diclofenac glucuronidation than those of intestine and kidney using tissue microsomes (Figure 4), suggesting that the liver is the major metabolic organ in vivo. Furthermore, we determined the kinetics differences in diclofenac glucuronidation across the tested species, indicating that the metabolism may not be translatable directly between animal and human models (Figure 6. Table 1).

In the UGT isoform study, we tested 12 major UGT isoforms from 1A and 2 B families. The results showed that UGT2B7 was the major isoform metabolizing diclofenac into its glucuronide, where the metabolic rate was almost two-fold that of other isoforms (Figure 3). Our results were consistent with the literature wherein UGT2B7 was compared with UGT1A6, 1A9, and 2B1 [17]. Glucuronidation in the acyl group has been reported to be mainly catalyzed by the UGT2B family for several other drugs such as mycophenolic acid [18]. We then tested other two isoforms from 2B family, namely, UGT2B4 and 2B10, which have high expression levels in the liver, intestine, and/or kidney. The results showed that UGT2B4 and 2B10 could also mediate glucuronidation of diclofenac, but the rates were similar to those of UGT1As (Figure 3).

In the tissue microsome study, we found that the metabolic rates with liver microsomes were significantly higher than those of intestine and kidney microsomes in both rats and humans. A recent protein analysis also showed that in humans, UGT2B7 is mainly expressed in the liver compared to other organs [19]. These findings could explain why the liver glucuronidation rate was significantly higher than that of the intestine and kidney in both rat and human microsomes (Figure 4). The result also revealed that UGT2B7 in the liver facilitates diclofenac enterohepatic recycling via glucuronidation.

The glucuronidation difference across species was initially evaluated using a single substrate concertation at 10 μM by incubating the substrate with liver microsomes of humans, monkeys, dogs, rats, and mice. The results showed that the metabolic rate in rat microsomes was significantly lower than those in the other species (Figure 5). Hence, extrapolation using rat data for human doses may lead to toxicity from the reactive acyl glucuronide due to this reactive metabolite’s higher rate of formation in humans as compared to rats. It may be more practical to carry out a wider range of cross-species metabolic studies for each compound before translation to human doses or establishing PK/PD models from animal data. Kinetic studies further reveal that the V_max_, K_m_, and Cl_int_ are highly different across these species in terms of diclofenac glucuronidation (Figure 6, Table 1). Species difference in glucuronidation has previously been reported in other compounds, such as regorafenib [20], glabridin [21], and wogonin [22], which may affect the correlation between animals with humans in terms of pharmacology and toxicity. Others have observed similar kinetic behaviors in diclofenac and ibuprofen glucuronidation in human liver microsomes that can be closely replicated in hUGT1 mouse liver microsomes, especially for drugs that are substrates of UGT1 enzymes [23]. Comparing with the reported data, it could be concluded that species difference in glucuronidation is compound-dependent. For example, for wogonin, the Cl_int_ in dog liver microsomes is the smallest across dog, rat, mouse, monkey, and human liver microsomes. In this study, we found that for diclofenac the Cl_int_ is the fastest in dog liver microsomes among those same species [22]. A literature search showed that many pharmacokinetic, pharmacological, and toxicological studies of diclofenac were on small and large preclinical animal models [23,24,25,26,27]. Our findings lead to a critical question: Are these models appropriate? When considering the glucuronidation aspect of diclofenac studies, the mouse appears to be a superior choice compared to the rat among the commonly available and affordable animal models. Since diclofenac also undergoes phase I metabolism mediated by CYP [28], further studies determining interspecies differences in its phase I metabolism would also be valuable.

These findings indicated that there are potential interspecies differences that may have implications for diclofenac metabolism and cross-species extrapolation in preclinical research. This may be due to differences in genetic makeup and UGT isoform expression levels, and catalytic activities of drug-metabolizing enzymes in these species. There has been a strong link between UGT2B17 levels and the formation of diclofenac glucuronide (DG) in intestinal microsomes, indicating UGT2B17′s significant role in the intestine. However, in the liver, DG formation correlated more closely with UGT2B7 expression, as UGT2B7 is more abundantly present in this organ, highlighting its primary role in hepatic glucuronidation [29]. There have also been several reports of intraspecies differences, which holds true for the same reason. This emphasizes the need for the cautious extrapolation of results from animal models to humans in drug development and safety assessments.

There are only in vitro studies using tissue microsomes and UGT supersomes in this study. The kinetic values, including V_max_, K_m_, and Cl_int_, may not accurately reflect the in vivo disposition of diclofenac, as it has been reported that diclofenac and its glucuronide are a substrate of OAT1/3 transporters [14]. Additionally, as mentioned above, phase I metabolism is also highly involved in the disposition of diclofenac. To fully elucidate the mechanism of clearance in vivo in different species, a quantitative comparison of the contribution of glucuronidation and phase I metabolism of diclofenac would be necessary. Although sex-dependent differences in metabolism have been linked to a variety of drugs, King and others [14] did not observe any significant difference in the glucuronidation of diclofenac between male and female human liver microsomes. As such, we did not assess sex-dependent differences in diclofenac glucuronidation in this paper because the focus of this study was to investigate the species- and tissue-dependent differences in the glucuronidation of diclofenac, making such comparative analysis beyond the scope of this study.

## 4. Materials and Methods

### 4.1. Chemicals and Materials

Diclofenac (purity 98%) and diclofenac acyl-β-D glucuronide (purity ≥ 95%) were purchased from Santa Cruz Biotechnology Inc., (Dallas, TX, USA). Alamethicin and uridine diphosphate glucuronic acid (UDP-GA) were purchased from Sigma-Aldrich (St. Louis, MO, USA). Methanol and acetonitrile (HPLC-grade) were obtained from VWR International (Radnor, PA, USA). Saccharolactone, rutin, formic acid, and magnesium chloride were purchased from Sigma-Aldrich (St. Louis, MO, USA). All other chemicals and solvents were used as received.

### 4.2. UGT Isoforms

Human recombinant UGT 1A1, 1A3, 1A4, 1A6, 1A7, 1A8, 1A9, 1A10, 2B4, 2B7, 2B10, and 2B17 supersomes with stock concentrations of 5 mg/mL were purchased from Corning Discovery Labware Inc., Woburn, MA, USA.

### 4.3. Tissue Microsomes

Microsomes (pooled) from male IGS rats (20 mg/mL), male cynomolgus monkeys (20 mg/mL), male CD1 mice (20 mg/mL), male beagle dogs (20 mg/mL), and male humans and tissues (liver—20 mg/mL; intestinal—10 mg/mL; and kidney—10 mg/mL) were purchased from Sekisui Xenotech (Kansas City, KS, USA).

### 4.4. Methods

#### 4.4.1. In Vitro Diclofenac Glucuronidation Reaction Using Microsomes

An in vitro phase II glucuronidation reaction was carried out using a published protocol that involves the use of uridine diphosphate glucuronic acid (UDPGA) as cofactor, saccharolactone as beta-glucuronidase inhibitor to drive the forward reaction to release the metabolite, potassium phosphate buffer (KPI) to mimic physiological conditions, and alamethicin as a surfactant for the reaction [30]. Based on our preliminary studies, the protein showed linearity with respect to time, and we found the appropriate concentrations to be 0.05 mg/mL for microsomes, 0.01 mg/mL for UGT supersomes, and 2 h incubation time at 37 °C with different substrate concentrations. All the samples and solutions were brought from the freezer and put on ice. The microsomes were pretreated with alamethicin for optimal activation at on ice for 15 min. The components were pipetted into Eppendorf tubes in the following order: substrate, KPI (pH 7.4), solution B (25 mM saccharolactone and 5 mM magnesium chloride), solution A (25 mM uridine diphosphate glucuronic acid and potassium chloride), microsome. The solution was preincubated at 37 °C for 5 min, after which the reaction was started by adding 20 µL of UDPGA for a total volume of 170 µL. Samples (100 µL) were taken at 2 h and the reaction was terminated with 50 µL of solution containing 0.6% formic acid in acetonitrile + internal standard (rutin) at each time point and vortexed for 1 min. The samples were then centrifuged at 14,000 rpm for 15 min. The supernatant (100 µL) was then collected and loaded into the UHPLC for analysis. The results were analyzed by determining the peak area ratios and plotting them against their respective concentrations obtained from a standard curve and analyzed using GraphPad Prism 10 software.

#### 4.4.2. Diclofenac Glucuronidation Using Different Tissue Microsomes

Diclofenac was employed as a substrate, and microsomes from rat tissues were utilized to measure the rate of the glucuronidation process. The final concentrations used for rat liver microsomes based on our preliminary studies was 0.05 mg/mL. The intestinal and kidney microsomes had a final concentration of 0.1 mg/mL. After addition of the components as described in the protocol, the mixture samples were all incubated at 37 °C for two hours with diclofenac concentrations at 10 μM, 20 μM, and 80 μM, respectively. The glucuronide metabolite was quantified using UHPLC and the metabolic rates were calculated accordingly.

#### 4.4.3. Diclofenac Glucuronidation with Different UGT Isoforms

An in vitro glucuronidation reaction was conducted using the same protocol involving the use of a cofactor (uridine diphosphate glucuronic acid (UDPGA)), a beta-glucuronidase inhibitor (saccharolactone) to facilitate the formation of glucuronide, and alamethicin for surfactant purposes. To mimic physiological conditions, potassium phosphate buffer (KPI) was used as the diluent in the reaction and diclofenac (20 μM) was incubated with the 12 isoforms. The final concentration of the UGT isoform was 0.01 mg/mL.

#### 4.4.4. Diclofenac Glucuronidation Reaction Using Different Species

Liver microsomes from different species (rat, monkey, dog, mouse, and human) were utilized to measure the rate of the glucuronidation process. The final concentrations used for the liver microsomes based on our preliminary studies was 0.05 mg/mL. After addition of the components as described in the protocol, the mixture samples were all incubated at 37 °C for two hours with diclofenac concentrations at 1.25 μM, 2.5 μM, 5 μM, 10 μM, 20 μM, 40 μM, 80 μM, 160 μM, and 200 μM, respectively. The peaks in UHPLC analysis were quantified and further analyzed for their reaction rates.

#### 4.4.5. Kinetic Analysis of Diclofenac Glucuronidation

Kinetic analysis was performed using GraphPad Prism software. The data were fitted to the Michaelis–Menten model using the equation, V = [V_max_ × S]/[K_m_ + S], where V is the velocity of the reaction, V_max_ is the maximum velocity, S is the concentration of the substrate, and K_m_ is the Michaelis–Menten constant. Intrinsic clearance (Cl_int_) was calculated by dividing the V_max_ by the K_m_ value for each species tested. Data were presented as the means of three independent kinetic studies using pooled microsomes.

#### 4.4.6. Ultra-High Performance Liquid Chromatographic (UHPLC) Method for Metabolite Quantification

A stock solution of diclofenac was prepared in dimethyl sulfoxide (DMSO) at 10 mM concentration. The standard curve samples were prepared by diluting the stock solution into 200 μM to 0.78 μM in KPI buffer containing 0.6% formic acid in acetonitrile and internal standard (Rutin 1.0 μM) to mimic similar conditions in the reaction. A Waters ACQUITY UHPLC H-Class PLUS System connected to a photodiode array (PDA) detector was used for the chromatographic separation, detection, and quantification of the analytes. The chromatographic conditions were optimized to achieve the best sensitivity, and peak resolution of the analytes and were as follows: mobile phase—formic acid (0.1%) in water and acetonitrile; stationary phase (column)—C18, 1.7 µm, 2.1 × 50 mm; column temperature: 45 °C; flow rate 0.45 mL/min; injection volume: 10 μL. Elution method: gradient with the following schedule: 0–0.5 min at 5% B; 0.5–1.0 min, 5–55% B; 1.0–3.0 min, 55–90% B; 3.0–3.5 min, 90–95% B; 3.5–4.0 min, 95–5% B; 4.0–5.0 min, 5% B. UV detection wavelength was set to 276 nm. The chromatographic method used for the quantification was well validated with a linearity range of 0.09–200 µM for both analytes. Calibration curves were generated for diclofenac and diclofenac acyl-β-D glucuronide; y = 18,934x + 6946.7, R^2^ = 1 and y = 9529.1x + 7258.6, R^2^ = 0.9996, respectively, with a lower limit of quantification (LLOQ) of 0.78 µM and limit of detection (LOD) of 0.09 µM. Quality control samples were tested at low (1.56 µM), mid (6.25 µM), and high (50 µM) concentrations in a single batch and over three different batches, after which the accuracy and precision was determined to be within 84–103% for all the analytes. Selectivity of the method was assessed by spiking 50 µL of ACN in 3.125 µM of the analytes to target the LLOQ, for which there was a similar peak area as compared to the LLOQ in the calibration standards.

### 4.5. Data Analysis

The Michaelis–Menten plot to determine enzyme activity and kinetics (Cl_int_, K_m_, and V_max_) was generated by GraphPad Prism 10 software. *t*-tests were used to compare the means of triplicate samples analyzed and these were used compare the differences between microsomes across the different species and tissues.

## 5. Conclusions

In conclusion, the data in this paper indicate that the liver plays a primary role in the glucuronidation of diclofenac, with the intestine and kidney also displaying some degree of metabolic activity. Furthermore, it is apparent that variations between species significantly influence the rate of glucuronidation of diclofenac. Therefore, pharmacokinetic, pharmacodynamic, and toxicological studies involving different species should take these glucuronidation differences into account to avoid inaccurate evaluations.

## Figures and Tables

**Table 1:** Kinetic parameters for diclofenac glucuronidation by different liver microsomes of humans, rats, monkeys, mouse, and dogs. Data expressed as mean ± SD (*n* = 3).

Species	K_m_ (µM)	V_max_ (nmol/mg/min)	Cl_int_ (µL/min/mg)	Kinetic Model
Human liver	59.50 ± 7.79	6.66 ± 0.33	0.12 ± 0.02	Michaelis–Menten
Rat liver	24.03 ± 4.29 *	0.83 ± 0.04 *	0.03 ± 0.01 *	Michaelis–Menten
Monkey liver	17.87 ± 2.59 *	3.88 ± 0.15 *	0.22 ± 0.01 *	Michaelis–Menten
Dog liver	41.45 ± 10.21 *	5.05 ± 0.42	0.13 ± 0.01	Michaelis–Menten
Mouse liver	91.85 ± 8.05 *	7.22 ± 0.28 *	0.08 ± 0.01 *	Michaelis–Menten

Each value represents the mean ± SD of triplicates experiments. * Superscripts show and indicate significant differences (*p* < 0.05, *t*-test) compared to those by human liver microsomes.

## Data Availability

The data that support the findings of this study are available from the corresponding author, S.G., upon reasonable request.
